# Melatonin–Nitric Oxide Crosstalk in Plants and the Prospects of NOMela as a Nitric Oxide Donor

**DOI:** 10.3390/ijms25158535

**Published:** 2024-08-05

**Authors:** Adil Hussain, Brekhna Faheem, Hyung-Seok Jang, Da-Sol Lee, Bong-Gyu Mun, Nkulu Kabange Rolly, Byung-Wook Yun

**Affiliations:** 1 Department of Agriculture, Abdul Wali Khan University Mardan, Mardan 23200, Pakistan; 2 Department of Applied Biosciences, College of Agriculture and Life Sciences, Kyungpook National University, Daegu 41566, Republic of Korea; 3 Department of Zoology, Abdul Wali Khan University Mardan, Mardan 23200, Pakistan; 4 Department of Environmental and Biological Chemistry, Chungbuk National University, Cheongju 28644, Republic of Korea

**Keywords:** melatonin, NO, NOMela, NO release kinetics, ROS, RNS

## Abstract

Melatonin regulates vital physiological processes in animals, such as the circadian cycle, sleep, locomotion, body temperature, food intake, and sexual and immune responses. In plants, melatonin modulates seed germination, longevity, circadian cycle, photoperiodicity, flowering, leaf senescence, postharvest fruit storage, and resistance against biotic and abiotic stresses. In plants, the effect of melatonin is mediated by various regulatory elements of the redox network, including RNS and ROS. Similarly, the radical gas NO mediates various physiological processes, like seed germination, flowering, leaf senescence, and stress responses. The biosynthesis of both melatonin and NO takes place in mitochondria and chloroplasts. Hence, both melatonin and nitric oxide are key signaling molecules governing their biological pathways independently. However, there are instances when these pathways cross each other and the two molecules interact with each other, resulting in the formation of N-nitrosomelatonin or NOMela, which is a nitrosated form of melatonin, discovered recently and with promising roles in plant development. The interaction between NO and melatonin is highly complex, and, although a handful of studies reporting these interactions have been published, the exact molecular mechanisms governing them and the prospects of NOMela as a NO donor have just started to be unraveled. Here, we review NO and melatonin production as well as RNS–melatonin interaction under normal and stressful conditions. Furthermore, for the first time, we provide highly sensitive, ozone-chemiluminescence-based comparative measurements of the nitric oxide content, as well as NO-release kinetics between NOMela and the commonly used NO donors CySNO and GSNO.

## 1. Introduction

Melatonin, or N-acetyl-5-methoxytryptamine, is a tryptophan derivative that was originally discovered in the bovine pineal gland [1]. In animals, melatonin plays an active role in regulating circadian rhythms, sleep, locomotor activities, body temperature, mood, and food intake, as well as sexual behavior and immune responses [2]. Melatonin was first isolated by Lerner et al. [3] from the pineal glands of cows in 1958. It is a hormone produced and released by the pineal gland in humans and most animals that are active during the day. A unique characteristic of melatonin is its diurnal fluctuations; that is, its secretion follows a circadian rhythm, with the highest levels in the blood occurring around 3 am to 4 am. The increase in daily melatonin secretion is associated with an increase in the desire to sleep about 2 h before the individual’s usual bedtime. This rhythmic release of melatonin is controlled by the body’s central circadian rhythm generator, called the “suprachiasmatic nucleus (SCN)”, which is located in the forward region of the brain called the hypothalamus [4]. Afterward, melatonin was reported in several other prokaryotes and eukaryotes, including plants.

In higher plants, melatonin was first detected in 1995 by the Dubbels and Hattori groups [5,6]. The magnitude of research on melatonin in plants is very limited compared to animals. The findings so far reveal that, in plants, melatonin functions in diverse physiological roles, from seed germination and longevity to plant growth regulation, circadian cycle, and photoperiodicity, which in turn affect flowering, leaf senescence, and postharvest fruit storage [7,8,9,10]. Melatonin is also involved in regulating plant resistance against pathogens [11], promotes root regeneration and embryogenesis, and curbs the damaging effects of toxic chemicals, salts, heavy metals, temperature fluctuations, and UV radiations [12,13,14]. Melatonin acts as an antioxidant, predominantly in the regulation of reactive nitrogen species (RNS) such as nitric oxide (NO) and peroxynitrite (ONOO^¯^), reactive oxygen species (ROS) like OH, H_2_O_2_, and other molecules, thus protecting plant tissues under stress [12,15]. In plants, the effect of melatonin is associated with various regulatory elements of the redox network through feedback mechanisms. Its roles in controlling plant stress acclimation are slowly emerging [16].

Nitric oxide (NO), a tiny molecule, was initially regarded as an air contaminant. This gas has since been revealed to exhibit an extensive variety of physiological roles in both plants and animals. In plants specifically, NO has been recognized as an important mediator in various physiological processes, like seed germination, flowering, leaf senescence, and different environmental stresses [17,18]. NO is one of the key signaling molecules and has both antioxidant and pro-oxidant properties. This dual action of NO is primarily determined by its local concentration and the mode of its spatial generation. Under biotic and abiotic stress, NO in plants may react with different redox molecules and regulate protein function through various mechanisms [19,20]. Moreover, nitric oxide plays a vital role in seed dormancy and germination, growth, development, and root morphogenesis, as well as regulation of stomata in plants [9,18,21]. It has been proven experimentally that NO influences chromatin remodeling [22]. Specifically, NO regulates histone acetylation via S-nitrosation and inhibits histone deacetylase (HDA) complex [22,23]. Hence, it appears that the effect of NO is dependent on cell chromatin status. In cells with high euchromatin entropy, NO acts as an oxidant, whereas in cells with regular euchromatin, it may act as an antioxidant because of different NO-mediated effects on auxin metabolism, where auxin acts as a main epigenetic regulator.

## 2. Biosynthesis and Metabolism of Melatonin

**Figure 1:**
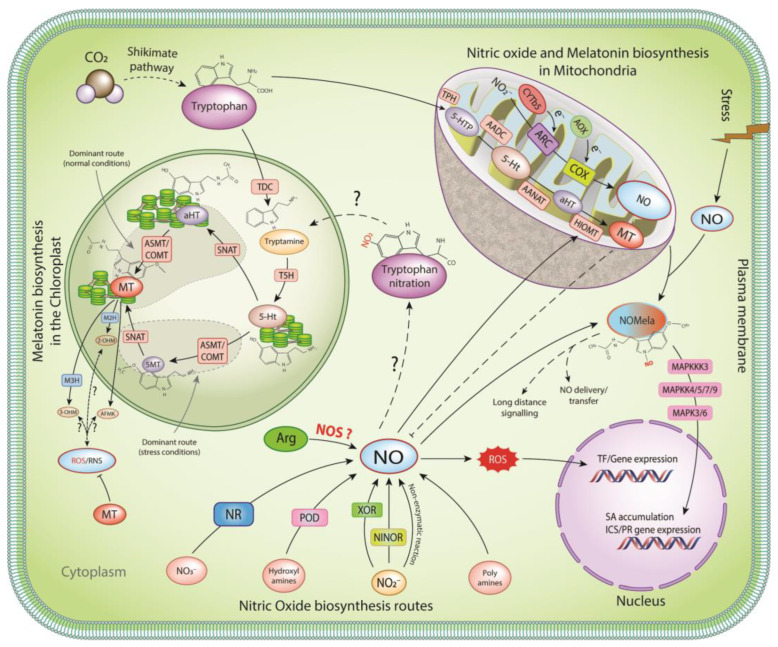
NO and melatonin production and interaction in plants.

In plants, the synthesis of melatonin takes place in mitochondria and chloroplasts [24,25] through the specialized route known as the shikimate pathway (Figure 1), involving at least six distinct enzymes [tryptophan decarboxylase (TDC), tryptophan hydroxylase, tryptamine 5-hydroxylase (T5H), SNAT, ASMT, and caffeic acid O-methyltransferase (COMT)] [26,27]. Tryptophan is decarboxylated to tryptamine, catalyzed by the TDC enzyme. This is followed by rapid hydroxylation via tryptamine 5-hydroxylase (T5H), a P450 enzyme found in the endoplasmic reticulum. Abiotic stress induces the expression of ASMT/COMT, resulting in the formation of 5-methoxytryptamine by the ASMT, followed by acetylation to form melatonin [27]. However, under normal conditions, the dominant pathway involves the acetylation of serotonin (5-Ht) by the SNAT enzyme to form N-acetyl-5-hydroxytryptamine (aHT) and then O-methylation to form melatonin [28]. Similarly, melatonin biosynthesis follows the same route in the mitochondria, where the AADC, AANAT, and HIOMT convert tryptophan to melatonin (Figure 1).

An important enzyme in melatonin degradation is melatonin 2-hydroxylase (M2H), an oxygenase enzyme that functions in the degradation of melatonin to its major metabolite, 2-hydroxymelatonin (2-OHM) [29]. M2H is located in the chloroplasts of the plant cells. Other important melatonin derivatives that are actively accumulated in plants include 3-hydroxymelatonin (3-OHM) catalyzed by melatonin 3-hydroxylase (M3H) in the cytoplasm [11], including cyclic melatonin, b-hydroxymelatonin, and cyclic b-hydroxymelatonin [30] (Figure 1). Although not clearly known, the generation of these metabolites may be due to the interaction of melatonin with oxidizing molecules, such as RNS and ROS [31].

Melatonin biosynthesis in plants occurs in mitochondria and chloroplasts [24] via the shikimate pathway, involving at least six distinct enzymes (tryptophan decarboxylase (TDC), tryptophan hydroxylase (TH), tryptamine 5-hydroxylase (T5H), serotonin N-acetyltransferase (SNAT), acetylserotonin O-methyltransferase (ASMT), and caffeic acid O-methyltransferase (COMT)) [27]. TDC converts tryptophan, which is hydroxylated by T5H, a P450 enzyme found in the endoplasmic reticulum. Stress induction of ASMT/COMT results in the formation of 5-methoxytryptamine, followed by acetylation to form melatonin [27]. Under optimal conditions, the dominant route involves the acetylation of serotonin (5-Ht) by the SNAT enzyme to form N-acetyl-5-hydroxytryptamine (aHT) and then O-methylation to form melatonin [28]. In mitochondria, aromatic L-amino acid decarboxylase (AADC), aralkylamine N-acetyltransferase (AANAT), and hydroxyindole O-methyltransferase (HIOMT) convert tryptophan to melatonin. Once formed, melatonin is converted to various metabolites, such as 2-OHM, 3-OHM, and N1-acetyl-N2-formyl-5-methoxykynuramine [AFMK]. Just like melatonin, NO is also produced in mitochondria and chloroplasts via multiple oxidative and reductive pathways [32]. NO production via the oxidation of L-arginine by a NO synthase-like enzyme and the oxidation of polyamine by polyamine synthase have been reported. Cytochrome oxidase and reductases generate NO from NADP and NADPH. Similarly, peroxidases convert N-omega hydroxyl-L-arginine (NOHA) to NO [33]. NO_3_^−^ are reduced to NO_2_^−^ by cytosolic nitrate reductase (NR), followed by NO via the action of xanthine oxidoreductase (XOR), plasma membrane-bound Nitrate-NO reductase (Ni-NOR), or NR in plastids [33]. NO production from nitrite occurs in mitochondria via the electron transport chain involving alternative oxidase (AOX) [34], the mitochondrial molybdopterin enzyme mARC, cytochrome b5 [35], and cytochrome-C oxidase (COX). NO and H_2_O_2_ levels rise during infection, leading to higher endogenous melatonin levels and activation of the MAPK cascade via OXI1/MAPKKK3, MAPKK4/5/7/9, and MAPK3/6 [36]. This activation of the MAP kinase cascade increases SA levels, inducing the expression of several defense-related genes, including PR1 [37]. The proximity of melatonin and nitric oxide in the same cellular organelles, as well as the published literature, suggests complex coordination between NO and melatonin to drive plant physiological responses under basal and stress conditions.

## 3. Relationship of Nitric Oxide and Melatonin in Plants

Just like melatonin, NO is known to be produced in mitochondria and chloroplasts. The NO production machinery of plants and animals has distinct differences, as NO production in plants occurs via multiple oxidative and reductive pathways [32]. One of the oxidative pathways leads to NO production via the oxidation of L-arginine by an NO synthase-like enzyme. Likewise, the oxidation of polyamine by polyamine synthase produces NO. Cytochrome oxidase and reductases can act upon NADP and NADPH, which leads to the production of NO. N-omega hydroxyl-L-arginine (NOHA) is converted to NO by peroxidase (POD) [33]. Alternatively, in the reductive pathway, nitrate reductase in the cytosol or plasma membrane reduces NO_3_^−^ to NO_2_^−^. In the following step, a group of enzymes like nitrate reductase in plastids, xanthine oxidoreductase (XOR), and plasma membrane-bound nitrate-NO reductase (Ni-NOR) contribute to the conversion of NO_2_^−^ to NO [33].

After decades of research, the most well-known route for the production of nitric oxide (NO) is perhaps the reduction of nitrite to NO through various nonenzymatic or enzymatic mechanisms. In plants, the primary enzymatic systems that carry out this reductive NO production are nitrate reductases (NRs), the mitochondrial electron transport chain [38], and a newly discovered complex between NR and NOFNiR (nitric oxide-forming nitrite reductase) in the unicellular alga *Chlamydomonas reinhardtii* [39]. Havemeyer, Bittner, Wollers, Mendel, Kunze, and Clement [39] showed that *C. reinhardtii* NR can interact with another partner protein from the amidoxime-reducing component (ARC) protein family, the NOFNiR, for the biosynthesis of NO from nitrite.

Mitochondria are an important source of NO in plants. NO production from nitrite can occur from electron pressure in the Q-cycle of Complex III. Alternative oxidase, which acts as a non-energy-conserving electron sink upstream of the Q-cycle, can reduce this electron pressure to produce NO [34]. The mitochondrial molybdopterin enzyme mARC reduces nitrite to NO using cytochrome b5 as an electron donor. The mARC proteins may represent a new pathway for hypoxic NO production in vivo [35] (Figure 1). However, cytochrome-C oxidase (COX) is both a source and a target of NO [40]. In addition to the reductive pathways, there is evidence to suggest the existence of an oxidative NO production route that is dependent on arginine, similar to the activity of nitric oxide synthase (NOS) in animals. However, no NOS homologs have been found in embryophyte genomes, and while there is increasing evidence for NOS-like activity in plants, the proteins involved have yet to be identified (Figure 1).

NO exerts its function via reversible posttranslational modification of cysteine thiols called S-nitrosothiols (SNOs). Depending on the cell chromatin status (stage/structure), NO can either be beneficial, promoting the survival and development of plants, or the opposite, as excessively high levels can cause oxidative injury in plant tissues. The global levels of SNOs in living organisms are thus maintained by the canonical antioxidant enzyme S-nitrosoglutathione reductase (GSNOR) [41].

### 3.1. NO, RNS, and Melatonin

As melatonin is produced in both animals and plants, Blask et al. [42] used the term phytomelatonin to differentiate between the two sources. Initially, it was shown to regulate plant growth, but is now known to play a key role in cell metabolism and responses to various biotic and abiotic stresses. It can act as a hormone as well as an antioxidant by scavenging various ROS and RNS. Furthermore, its amphiphilic characteristics make it easily permeable through membranes and enable it to move into the cytosol and organelles, further facilitating its regulatory role [43]. Melatonin and nitric oxide are both extremely important molecules required for normal physiology, governing their biochemical pathways independently. However, there are instances when these pathways cross each other and these two molecules interact with each other, resulting in the formation of *N*-nitrosomelatonin or NOMela. NOMela represents a nitrosated form of melatonin, discovered recently and with promising roles in plant physiology. The interaction between NO, other reactive nitrogen species (RNS), and melatonin exhibits a certain degree of complexity, since they act independently but also interact to regulate multiple signaling pathways [36]. However, extensive roles of phytomelatonin and NO in modulating various physiological pathways are emerging (reviewed by Mukherjee [16,44], Mukherjee [45]). Several studies [46,47,48,49,50,51] suggest interactions between phytomelatonin and NO or the putative plant NOS. Among the various S-nitrosated molecules, NOMela is emerging as an important signaling molecule in both animals and plants [52]. Aghdam et al. [53] reported that the exogenous application of melatonin promotes the accumulation of NO by triggering the activation of the arginine pathway in tomato fruits. Recently, Liu et al. [54] showed a significant delay in fruit senescence due to an increase in the NO content following melatonin application. On the other hand, the concentration of NO in tomato seedlings increases following the exogenous application of melatonin via the disruption of S-nitrosoglutathione reductase (GSNOR) activity and an increase in the expression of nitrate reductase (NR) [55].

Similarly, NO application increases the level of melatonin by inducing the expression of tryptophan decarboxylase (TDC), tryptamine 5-hydroxylase (T5H), serotonin N-acetyltransferase (SNAT), and caffeic acid O-methyltransferase (COMT) via a cyclic guanosine monophosphate (cGMP) pathway. The cyclic 3-hydroxymelatonin (C3HOM) is an immediate product of melatonin’s interaction with various reactive oxygen species (ROS). In addition, other forms of melatonin metabolites have also been reported in animals and plants, such as 3OHM, 2OHM, and N1-acetyl-N2-formyl-5-methoxykynuramine (AFMK—the first melatonin reported from plants [56]). These melatonin metabolites are also known to react with NO in the course of stress signaling. In this regard, 2-hydroxymelatonin (2OHM), in particular, could be an active molecule in the NO-mediated signaling pathways. Exogenous application of 2OHM enhances plant tolerance to cold, drought [57], and cadmium stress [58]. NO combines with melatonin to form *N*-nitrosomelatonin (NO-Mela) via nitrosation. NO can nitrosate melatonin via a radical mechanism or an ionic mechanism under different pH conditions. NO-Mela is an effective NO donor in cultured cells, especially in the presence of serotonin and its derivatives [59]. NO donor sodium nitroprusside (SNP) can mimic the effects of light on photoreceptor melatonin synthesis [60]. NO–melatonin crosstalk is particularly important during stress responses. Melatonin triggers NO accumulation via NR modulating the activity of nitrate reductase (NR) and nitric oxide synthase (NOS) enzymes [53,61,62]. However, NO-melatonin antagonistic interactions have also been described, such as NOS inhibition by melatonin [63]. This shows that melatonin can induce NO production but can also induce NO scavenging mechanisms. Thus, it is a key player in NO regulation at the cellular level.

### 3.2. NO, Melatonin, and Antioxidative Defense

Melatonin is fundamentally considered an antioxidant with an imperative role in regulating reactive nitrogen and reactive oxygen species. Melatonin has a high scavenging potential toward·OH, ·NO, ·NO2, ·ONOO-, and N3. A decline in oxidative damage has been observed in the animal system as a result of the inhibition of NO synthase activity and NO biosynthesis caused by the regulatory action of melatonin [63]. Furthermore, melatonin is capable of minimizing the detrimental effects of several xenobiotics in both plants and animal cells [9]. There are two underlying mechanisms involved in this process: (1) melatonin acts directly on RNS and ROS generated by the xenobiotics, and (2) melatonin acts indirectly to initiate the antioxidant enzyme gene expression, such as catalases, halo-peroxidases, glutathione-, glutathione synthases, glutathione reductases, glutathione S-transferases, ascorbate-, ascorbate oxidases, monodehydro- and dehydroascorbate reductases, thioredoxins, peroxiredoxins, etc., all geared towards curtailing ROS toxicity [9]. Likewise, the three principal melatonin derivatives, AMK, AFMK, and 3OHM, play an additional significant role in lowering oxidative distress in plants and animal systems [64,65]. Similarly, NO plays an important function in counteracting ROS-mediated damage during stress [66]. For instance, the application of NO to maize plants leads to a decline in the H_2_O_2_ content amid the activation of the antioxidative defense of maize. Similarly, in *Brassica juncea*, the exogenous NO significantly overcomes the oxidative injury produced by salt stress [67].

### 3.3. NO, Melatonin, and Stress Response

Plants produce variable levels of melatonin under different circumstances [12]. However, its production is increased under a variety of stress conditions, with its highest concentration in the flowers, followed by the leaves and seeds [68]. Information regarding the interaction of melatonin and NO in plants is limited. Only a handful of studies reporting these interactions have been published over the past decade, and the exact molecular mechanisms governing these interactions have just started to be unraveled. Nonetheless, melatonin treatment is known to alter endogenous NO levels in plants. A brief account of the published literature reporting NO–melatonin interaction under different physiological conditions is shown in Table 1. Melatonin is known to scavenge H_2_O_2_, OH, NO, ONOO^-^, HOCl, and ^1^O_2_, thereby providing protection under biotic and abiotic stress conditions [69]. Foliar treatment with melatonin protects *Silybum marianum* plants against salt stress by modulating photosynthesis and activating the antioxidant machinery [70]. NO, on the other hand, may act downstream of melatonin to promote plant defense against a variety of biotic and abiotic stresses [71].

Recently, Khan et al. [72] showed that melatonin-regulated heat shock proteins and mitochondrial ATP synthase confer drought tolerance via the maintenance of ROS homeostasis in a hydrogen sulfide-dependent manner, whereas [73] reported melatonin-mediated enhancement of photosynthesis and salt tolerance in wheat. Similarly, the exogenous application of melatonin has been shown to mitigate chilling stress in mango [74], drought tolerance in tomato [75], salt stress in *Ranunculus asiaticus* [76], polyethylene (PEG)-induced osmotic stress in soybean [77], salt stress in tomato plants via modulation of carbohydrate and nitrogen metabolism [78], waterlogging stress in *Zanthoxylum armatum* via enhancement of photosynthesis and increase in carotenoid content [79], and to protect rice plants against rice blast disease by acting synergistically with the fungicide isoprothiolane to interfere with lipid metabolism by targeting the isocitrate lyase-encoding gene MoICL1 [80]. Xu et al. [81] reported that exogenous melatonin application protected the ornamental woody plant rhododendron against extended heat stress by improving the melatonin contents, electron transport rate, photosystem II and I activities, rubisco activity, and ATP content. Furthermore, they performed transcriptomic analysis to identify several heat-induced differentially expressed genes associated with photosynthetic activity.

One very interesting and recent study (preprint) highlighted a key aspect of the melatonin and nitric oxide interaction in tomato, where Gong et al. [82] showed that saline-alkali stress induced the S-nitrosation of the plasma membrane H^+^-ATPase 2 (HA2) at Cys206 aggravated by GSNOR knockdown but alleviated by COMT-overexpression. The COMT was found to enhance melatonin synthesis and NO scavenging to improve saline-alkali tolerance. HA2 S-nitrosation suppressed its interaction with the 14-3-3 protein 1 (TFT1), resulting in the inhibition of its enzymatic activity and saline–alkali tolerance. This indicates that melatonin relieves the S-nitrosation of plasma membrane H^+^-ATPase 2 to enhance saline–alkali tolerance in tomato. They concluded that, under physiological status, melatonin and NO act jointly as a redox switch of HA2 to regulate root H^+^ and Na^+^ efflux to affect saline–alkali tolerance. Once published, this may be the first convincing study showing the molecular basis of melatonin and nitric oxide interaction under saline–alkali stress in tomato through the melatonin-HA2-S-nitrosation module.

Endogenous melatonin content in plants varies with circadian rhythms, indicating a central role for melatonin in plant development [10]. The melatonin receptor CAND2 mediates H_2_O_2_ and Ca^2+^ signaling to regulate melatonin-induced stomatal closure in Arabidopsis [83]. Exogenous application of melatonin increases endogenous NO levels via inhibition of GSNOR activity [55]. Corpas et al. [84] suggested that melatonin accumulation may act as a NO scavenger during tomato fruit ripening. Similarly, nitrosated melatonin (NOMela) mediates the crosstalk between NO and ethylene during fruit ripening [45]. NO–melatonin interaction regulates a plethora of plant responses under a variety of stress conditions (reviewed by Mukherjee and He and He [85]).

In plants, melatonin may be implicated in day–night cycles. Plants maintain a balance between melatonin biosynthesis and degradation. However, the concentration of melatonin may increase in response to environmental stress. Furthermore, information regarding the concentration of melatonin during various growth stages is largely unknown. For instance, Arnao and Hernandez-Ruiz [86] reported that the levels of melatonin in tomato plants in vitro were highest in leaves followed by roots and stem, whereas, in the open field, the melatonin content of tomato plants was in the order of leaf > stem > root. This may have been the result of variations in the duration and intensity of light in the field and in controlled conditions. Likewise, it is induced by NaCl in barley roots [87]. High-intensity UVB radiation for a short period was also found to induce a greater concentration of melatonin in *Glycyrrhiza uralensis* roots [88].

Melatonin is a multifunctional signaling molecule with diverse roles in plant physiology. These roles are often orchestrated via intricate networks that influence other phytohormones [89,90]. For example, similar to auxin, melatonin promotes root generation and lateral/adventitious roots [91]. Melatonin is also known to affect gibberellin metabolism and cytokinin levels and modulate the expression of ABA and ethylene pathway genes, thereby affecting fruit ripening and postharvest processes [92,93]. Melatonin also contributes to plant immunity alongside salicylic acid (SA) and jasmonic acid (JA). Studies have shown that melatonin protects chlorophyll and promotes cellular integrity by delaying senescence [92,93]. On the other hand, the role of NO in regulating phytohormones is well known. Several key proteins involved in phytohormone signaling are known to be modulated by NO via S-nitrosation. For example, the S-nitrosation of ABI5 triggers its degradation, promoting seed germination and seedling growth [94]. COP1 S-nitrosation inhibits its function during photomorphogenesis [95]. The auxin receptor transport inhibitor response 1 (TIR1) undergoes S-nitrosation, which enhances its interaction with Aux/IAA to form a coreceptor crucial for auxin responses in plants [96]. Similarly, other proteins, such as JAZ (JA pathway) and NPR1 (SA-pathway) proteins, are known to be regulated by NO via S-nitrosation [97]. As recent studies (including this one) propose NOMela as a new and versatile NO donor, it would be interesting to explore the impact of this new NO donor on phytohormonal networks in plants.

Plants are frequently exposed to phytopathogenic bacteria, viruses, fungi, and herbivores. Melatonin is emerging as a plant defense regulator, as the exogenous application of melatonin improves the resistance of *Malus prunifolia* to Marssonina apple blotch [98]. The expression of defense genes *PR1*, *ICS1*, and *PDF1.2* decreased after infection with the avirulent pathogen *Pseudomonas syringae* pv. tomato (*Pst*DC3000-*avr*Rpt2) [99]. External application of melatonin increased plant resistance to pathogens such as *Fusarium oxysporum*, *Penicillium* spp., *Phytophthora infestans*, *Botrytis cinerea*, and *Rhizopus stolonifera* [100]. In cotton, the external application of melatonin increased the expression of genes involved in the phenylpropanoid, mevalonate (MVA), and gossypol pathways following *Verticillium dahliae* inoculation [101]. As described, NO is a signaling molecule that plays a key role in plant responses to pathogen invasion and is upstream of the innate immune system in plants. Many studies have investigated NO-mediated plant disease responses and the relationship between NO and SA [19]. Plants deficient in SA (for example, the NahG-overexpressing line) and NO (*noa1* and *nia1nia2* mutant lines) are highly sensitive to bacterial pathogens. Both SA and NO regulate Arabidopsis resistance to pathogenic bacteria, with synergy between the two compounds playing an important role in natural immunity [102]. Treatment of Arabidopsis with melatonin followed by *Pseudomonas syringe* pv. tomato (*Pst*) DC3000 infection rapidly increased NO levels in leaves [103].

After infection of Arabidopsis with *Pst*DC3000, melatonin treatment induces transcription of CBF/DREB1s, leading to increased accumulation of soluble sugars [104] with an increase in the endogenous soluble sugars, glycerol, SA, and NO, resulting in enhanced resistance against pathogens [105]. Infection by pathogenic bacteria may also cause increased levels of H_2_O_2_ and NO, leading to enhanced endogenous melatonin level activation of the MAPK cascade via OXI1/MAPKKK3, MAPKK4/5/7/9 and MAPK3/6 [36]. This melatonin-mediated MAPK cascade activation has been shown to increase SA levels, inducing the expression of several defense-related genes, including PR1 [37] (Figure 1). From the above literature, it is clear that melatonin plays a vital role in the regulation of RNS, ultimately affecting physiological processes under stress. Furthermore, complex coordination between NO and melatonin drives plant responses against pathogen invasion. Further studies are required to understand the relationship between melatonin and NO via genomics, proteomics, transcriptomics, metabolomics, PTMomics, interactomics, proxiomics, etc.

**Table 1:** Nitric oxide–melatonin interaction in plants.

S. No.	Plant Species/Tissue	Plant Physiological Processes	NO Content	Melatonin Content	References
**Biotic stress**					
1	Arabidopsis	Pathogen infection	+	+	[37,102,105]
2		Bacterial infection	+	+	[102,103,105]
3	Tomato	Pathogen infection	+	+	[106]
**Abiotic stress**					
4	Tomato	Cold stress	+	+	[53]
5	Capsicum	Salt stress	+	+	[107]
6	Alfalfa	Drought stress	+	+	[61]
7	Sunflower	Salt stress	+	+	[108,109]
8	Rapeseed	Salt stress	+	+	[110]
9	Arabidopsis	Aluminum/Cd toxicity	+	−	[111]
10		Iron deficiency	+	+	[112]
11	Wheat	Aluminum/Cd toxicity	+	+	[113]
12	Cucumber	Cold stress	+	+	[114]
**Growth and Development**					
13	Tomato	Root development	+	+	[55]
14		Response to alkalinity	+	+	[115]
15		Saline-alkali stress	+	+	[82]
16	Capsicum	Fruit ripening	+	+	[44]
17	Pear	Fruit senescence	+	+	[54]

+ = increase. − = decrease.

### 3.4. NOMela as a Nitric Oxide Donor

The roles and effects of nitric oxide and melatonin have been established, and much research has been conducted over the past few decades on the use of these important signaling molecules, especially nitric oxide, in both animal and plant systems. However, research involving N-nitrosomelatonin (NOMela) is very limited. NOMela is well known for its ability to transnitrosylate nucleophiles, including ascorbate and thiols [116]. Blanchard-Fillion et al. [117] showed that NOMela releases NO, thereby acting as an efficient NO donor. Afterward, other studies have reported the shift of NO moiety from NOMela to other biological macromolecules, including various activated hydroxy compounds like vitamin C [118,119], vitamin E [120], catechol [121], serotonin [59], and protein thiols [122]. Berchner-Pfannschmidt, Tug, Trinidad, Becker, Oehme, Flamme, Fandrey, and Kirsch [116] used NOMela as a NO donor in cell culture experiments and recorded significantly higher viability of the cells, likely due to the antioxidant properties of NOMela. From studies in animal systems, it appears that NOMela has prominent melatonin-like effects, as NOMela is known to enhance photic synchronization of mammalian circadian rhythms correlated with high suprachiasmatic immunoreactivity of the proto-oncogene cFOS and period circadian regulator 1 (PER1) [123]. In animals, NOMela and the melatonin derivative NOM (1-nitroso melatonin) are NO as well as melatonin donors and are considered new potential drugs, particularly in neurological diseases [124].

As described earlier, studies involving the use of NOMela as an NO donor are very limited, particularly in plant sciences. Recently, Singh et al. [125] reported a preferential uptake of NOMela by Arabidopsis roots compared to the commonly used NO donor GSNO. They also recorded a long-distance transport of NOMela via the vascular bundles, releasing 52.8% more NO compared to GSNO. Using confocal laser scanning microscopy, they showed a strong NO signal generated in the mitochondria. They concluded that NOMela is a more efficient NO donor than an equimolar concentration of GSNO. Apart from this finding, no other studies have been published involving the use of NOMela in plants. One interesting reason for this (at least in our hands) is the apparent difficulty in synthesizing N-nitrosomelatonin, and the commercially available stocks are currently too expensive. Melatonin can be converted to NOMela in the presence of oxygen and acidic pH conditions [119,126]. Melatonin and nitric oxide are both produced in the same cellular compartments, and there is considerable evidence for the physical and chemical interaction between the two signaling molecules [53,61,107,108,109,110,113,115,127,128].

**Figure 2:**
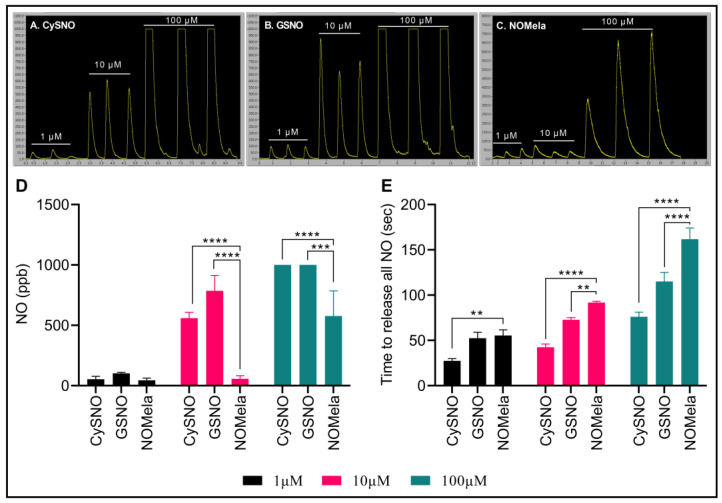
Comparison of CySNO, GSNO, and NOMela as NO donors. (**A**–**C**) Comparative ozone-chemiluminescence-based comparative NO measurements via Sievers NA 280i NO Analyzer. (**D**) Comparison of the nitric oxide content (ppb) in 100 µL of equimolar concentrations (1 µM, 10 µM, and 100 µM) of CySNO, GSNO, and NOMela. (**E**) Kinetics of NO released from 100 µL of equimolar concentrations of CySNO, GSNO, and NOMela. Each data point represents the mean of three independent replications, error bars represent standard deviation, and asterisks represent significant differences among the means determined via student’s *t*-test at *p* = 0.05 (**), 0.01 (***), and 0.001 (****) in Microsoft Excel. Standard and sample solutions were prepared, and NO measurements were performed in an NA280i NO Analyzer with a CuCl_2_ reducing buffer system, as described by Hussain, Yun, and Loake [129].

Here, we synthesized NOMela according to Kirsch and De Groot [52] at 4 °C in the dark and measured its NO-releasing capacity efficiency compared to the commonly used NO donors CySNO and GSNO using highly sensitive, ozone-chemiluminescence-based comparative measurements through the Sievers NA280i NO Analyzer with a CuCl_2_ reducing buffer system [129]. We compared three different concentrations of NOMela (1 µM, 10 µM, and 100 µM) with equimolar concentrations of CySNO and GSNO. Interestingly, our results indicated that NOMela released significantly less NO compared to GSNO and CySNO (Figure 2A–D). More precisely, 1 µM NOMela released an average of 16.9% and 56.8% less NO than equimolar concentrations of CySNO and GSNO, respectively. This pattern was more pronounced in the higher concentrations, where 10 µM NOMela released an average of 90% and 72.9% less NO than equimolar concentrations of GSNO, and CySNO, respectively. However, it was interesting to note that, under the same equimolar concentrations of the NO donors and reducing buffer conditions, NOMela took a significantly longer time to release all the NO than CySNO and GSNO (Figure 2A–C,E). More precisely, the time taken for complete reduction and release of NO from 100 µL of NOMela was approximately double the time taken by the same volume of equimolar concentrations of CySNO and GSNO. This may be a highly important attribute of NOMela, particularly as an NO donor, as the slow and consistent release of NO can have long-lasting effects under physiological conditions. Considering this aspect of NOMela as an NO donor, our results corroborated with the findings of Singh, Jain, Gupta, Khurana, and Bhatla [125] who described NOMela as a better NO donor than GSNO. To our knowledge, this is the first report of highly sensitive ozone-chemiluminescence-based measurement of NO from NOMela. More work is needed to understand the rates of absorption, diffusion, release of NO and melatonin, and chemical kinetics of the newly discovered signaling molecule NOMela and its interaction with other components, such as phytohormones, metabolites, ROS, and RNS.

## Data Availability

All relevant data are available within the manuscript.
